# Mortality and glycemic control among patients with acute and chronic myeloid leukemia and diabetes: a case–control study

**DOI:** 10.2144/fsoa-2020-0117

**Published:** 2020-10-26

**Authors:** Julia E Wiedmeier, Luke J Mountjoy, Matthew R Buras, Heidi E Kosiorek, Kyle E Coppola, Patricia M Verona, Curtiss B Cook, Nina J Karlin

**Affiliations:** 1 Department of Internal Medicine, Mayo Clinic Hospital, 5777 E Mayo Blvd, Phoenix, AZ 85054, USA; 2 Division of Hematology & Medical Oncology, Mayo Clinic Hospital, 5777 E Mayo Blvd, Phoenix, AZ 85054, USA; 3 Colorado Blood Cancer Institute, 1721 E 19th Ave, Suites 200-300, Denver, CO 80218, USA; 4 Biostatistics, Mayo Clinic, 13400 E. SheaBlvd., Scottsdale, AZ 85259, USA; 5 Mayo Clinic Cancer Center, Mayo Clinic, 13400 E. Shea Blvd., Scottsdale, AZ 85259, USA; 6 Enterprise Technology Services, Mayo Clinic Hospital, 5777 E Mayo Blvd, Phoenix, AZ 85054, USA; 7 Division of Endocrinology, Mayo Clinic, 13400 E. Shea Blvd., Scottsdale, AZ 85259, USA

**Keywords:** cancer, endocrinology, outcomes research

## Abstract

**Aim:**

We examined the association between diabetes and survival in patients with acute and chronic myeloid leukemia and the association of leukemia with glycemic control.

**Patients & methods:**

Patients with leukemia with and without diabetes (2007–2015) were retrospectively identified and matched 1:1 (n = 70 per group). Overall survival was estimated by the Kaplan–Meier method. Hemoglobin A_1c_ and glucose levels the year after leukemia diagnosis were compared by mixed models.

**Results:**

Among 25 of 70 patients with diabetes, mean hemoglobin A_1c_ during the year after leukemia diagnosis was 6.8%. Kaplan–Meier-estimated 3-year survival was 46% for diabetes patients versus 45% for controls (p = 0.79).

**Conclusion:**

No associations were found between leukemia, diabetes, survival and glycemic control.

## Lay abstract

The goal of this study was to evaluate the association between diabetes and survival among patients with acute and chronic myeloid leukemia and the association between leukemia and glycemic control. A total of 140 patients with acute and chronic leukemia were retrospectively identified (70 with diabetes; 70 without diabetes). In this study, diabetes was not associated with decreased survival in patients with leukemia and leukemia was not associated with poor glycemic control.

Leukemia is one of the ten most common causes of cancer deaths in the USA [1]. It has a higher incidence in White persons and males. A common comorbid condition in many people with and without cancer is diabetes, present in an estimated 9.4% of the US population or 30.3 million people, as of 2017 [2]. Studies have shown an increased risk of death of certain solid cancers in patients with diabetes [3,4], but data are limited and inconsistent regarding the association between leukemia, diabetes and death.

The presence of diabetes in patients with cancer could conceivably lead to worse cancer prognosis. There are several possible mechanisms for the association between cancer and diabetes. Diabetes is a state of insulin resistance leading to abnormal glucose utilization and disturbances of glucose metabolism, which leads to chronic hyperglycemia [5] and cancer cells consume more glucose than normal cells [6]. Increased levels of circulating glucose allows malignant cells more access to glucose and thus an environment that gives cancer cells the nutrients to thrive and grow. Patients with diabetes often have increased levels of circulating insulin, which may also increase growth and proliferation of cancer cells [7]. Obesity and diabetes are chronic states of inflammation, which has also been shown to promote tumor growth and survival [8].

A large prospective cohort study in the US evaluated diabetes as a predictor of cancer death and found a significant association with solid organ cancers, but there was no significant relationship in diabetes patients with leukemia [9]. Another prospective cohort study among South and East Asians did not show an increased risk of death in diabetes patients with leukemia [10]. A retrospective analysis found an increased 30-day mortality rate in patients with diabetes among older adults with acute myeloid leukemia (AML) receiving intensive therapy [11].

We have previously published case–control studies showing no association between diabetes and decreased survival in patients with melanoma, endometrial cancer, pancreatic cancer, colorectal cancer or lung cancer, but we did show an association between death and progression in patients with diabetes and esophageal and gastric cancer [12–17]. Given the paucity of studies examining the relationship between diabetes and hematologic cancers, we aimed to investigate whether the presence of diabetes was related to higher mortality rates in patients with leukemia. Moreover, few studies have evaluated for an association between leukemia and glycemic control among patients with diabetes. For this analysis, we focused on patients with AML and chronic myeloid leukemia (CML).

## Methods

### Case selection

Institutional review board approval was obtained for this retrospective case–control study. We searched our institutional cancer registry for the records of adult patients (>18 years) with newly diagnosed AML or CML who were seen between 1 January 2007 and 31 December 2015. Cancer cases were linked to the electronic health record to identify patients with a diagnosis of diabetes using the International Classification of Diseases, Ninth Revision diagnostic code 250.00, as previously described [12–16]. Electronic health record data were examined around the time of cancer diagnosis to identify patients who had diabetes claims during this period. Data were collected regarding age at leukemia diagnosis, diagnosis date, ethnicity, BMI, allelic variation/translocation status of leukemia and treatment. In addition, we collected data on marital status, tobacco use, alcohol use, payer type, Eastern Cooperative Oncology Group performance status, corticosteroid use and transplant status. We chose to evaluate myeloid leukemias because myeloid and lymphoid leukemias are different entities based on precursor cells and lymphoid leukemias warrant a separate study.

We then cross-referenced these data against a list of all patients seen during the same period who had a diagnosis of Type 1 or Type 2 diabetes to categorize patients with leukemia by diabetes status (with or without diabetes). Patients who had another primary cancer were excluded. From this dataset, patients with leukemia and diabetes were matched 1:1 via a greedy matching algorithm to control patients with leukemia without diabetes. Variables included in the matching algorithm were sex and age at leukemia diagnosis. Patients were excluded if no chart review was able to be conducted for their matched pair or if the patient’s diabetes status could not be verified from the chart review.

Glucose and hemoglobin A_1c_ (HbA_1c_) values were derived from the laboratory information system. We then reviewed the electronic health record for additional detailed information on leukemia treatment (chemotherapy, targeted therapy, radiation therapy) and data related to diabetes (date of diabetes diagnosis, type of diabetic therapy and diabetic complications).

### Statistical analysis

Patients with CML or AML with diabetes (cases) and without diabetes (controls) were compared on the basis of patient characteristics and clinical variables. Continuous variables were compared by using paired *t* tests; categorical variables were compared by using the McNemar test or Bowker test for symmetry. HbA_1c_ levels during the first year after leukemia diagnosis were evaluated with a linear mixed model in the diabetes group only (HbA_1c_ values were unavailable for most patients without diabetes). Time was considered a fixed effect, and an individual specific random effect was included. A similar approach was used for modeling glucose values during that year. Fixed effects included time in days, case or control designation, an interaction term (days × case–control designation) and patient-specific and matched pair-specific effects. Glycemic control was defined as a mean glucose value less than 126 mg/dl during the first year after cancer diagnosis.

Overall survival (OS) was defined as the time from leukemia diagnosis until death of any cause. For OS, patients were considered censored at the last known follow-up date if death was not documented in the health record. Three-year OS was estimated with the Kaplan–Meier method and compared between groups by using the log-rank test. Cox proportional hazards regression was used to assess for effect of diabetes and included OS, with matched pairs as the strata variable. Sample size was based on the number of available cases in 2007–2015; it provided 80% power to detect a difference of 15% in 3-year survival rate estimates between cases and controls. p-value less than 0.05 was considered statistically significant; SAS version 9.4 (SAS Institute Inc., NC, USA) was used for analysis.

## Results

### Patients with leukemia

The original matched dataset contained 83 pairs; after exclusion of 13 pairs per the stated criteria, 70 matched pairs (N = 140) were included in the analysis (Table 1). The median age of patients at leukemia diagnosis was 62 (range: 19–94) years, 60% were men and 89% had AML. The majority of patients were White (83.6%) and non-Hispanic (60.0%) (Table 1).

**Table 1.** Patient characteristics^†^.

Characteristic^‡^	Total (n = 140)	Group		p-value
		Diabetes (n = 70)	No diabetes (n = 70)	
Age at diagnosis, y	55.3 (16.0)	55.3 (16.3)	55.2 (15.8)	NA
Women	56 (40.0)	28 (40)	28 (40)	NA
Ethnicity				0.98
Hispanic	24 (17.1)	12 (17)	12 (17)	
Non-Hispanic	84 (60.0)	43 (61)	41 (59)	
Unknown	32 (22.9)	15 (21)	17 (24)	
Race				0.16
White	117 (83.6)	57 (81)	60 (86)	
American Indian/Alaskan native	6 (4.3)	6 (9)	0	
Asian	5 (3.6)	1 (1)	4 (6)	
African–American	4 (2.9)	2 (3)	2 (3)	
Other	6 (4.3)	3 (4)	3 (4)	
Unknown	2 (1.4)	1 (1)	1 (1)	
Leukemia type				0.60
Chronic	16 (11.4)	9 (13)	7 (10)	
Acute	124 (88.6)	61 (87)	63 (90)	
Acute leukemia subtype	(n = 96)	(n = 36)	(n = 60)	0.01
Favorable	14 (14.6)	5 (14)	9 (15)	
Intermediate	33 (34.4)	6 (17)	27 (45)	
Unfavorable	49 (51.0)	25 (69)	24 (40)	
Variation/translocation^§^	(n = 113)	(n = 52)	(n = 61)	0.001
None	43 (38.1)	10 (19)	33 (54)	
Translocation	40 (35.4)	26 (50)	14 (23)	
Variation	30 (26.5)	16 (31)	14 (23)	
Transplant	67 (51.5) (n = 130)	38 (57) (n = 67)	29 (46) (n = 63)	0.22
GVHD	52 (78.8) (n = 66)	31 (82) (n = 38)	21 (75) (n = 28)	0.52
BMI category				0.28
<25.0	51 (36.4)	21 (30)	30 (43)	
25.0–29.9	27 (19.3)	15 (21)	12 (17)	
≥30.0	62 (44.3)	34 (49)	28 (40)	
Any alcohol use				0.59
Yes	51 (36.4)	23 (33)	28 (40)	
No	84 (60.0)	44 (63)	40 (57)	
Unknown	5 (3.6)	3 (4)	2 (3)	
Smoking status				0.74
Never	70 (50.0)	36 (51)	34 (49)	
Former	56 (40.0)	28 (40)	28 (40)	
Current	9 (6.4)	3 (4)	6 (9)	
Unknown	5 (3.6)	3 (4)	2 (3)	
Use of corticosteroids	45 (36.0) (n = 125)	44 (79) (n = 56)	1 (1) (n = 69)	<0.001

† Values are mean (standard deviation) or number of patients (%).

‡ All values except transplant and GVHD are at the time of leukemia diagnosis.

§ In patients with acute leukemia only.

GVHD: Graft-vs-host disease; NA: Not applicable, matched variable; SD: Standard deviation.

Of AML and CML patients with data available, 79% of those with diabetes received corticosteroids, compared with 1% of those without diabetes (p < 0.001), and the proportion with graft-versus-host disease was similar between patients with and without diabetes (p = 0.52) (Table 1). Chemotherapy regimens were variable, with approximately 11 different conditioning regimens, at least 20 chemotherapy regimens and 15 different targeted therapy regimens (data not shown). Approximately 80% of patients with (n = 55) and without (n = 54) diabetes received some type of chemotherapy, whereas 11 patients without diabetes (16%) and 15 patients with diabetes (21%) had targeted therapy (data not shown). There was no significant difference in treatment regimens between patients with and without diabetes (p > 0.99).

### Patients with diabetes

#### Glucose control & treatment characteristics

**Figure 1.**
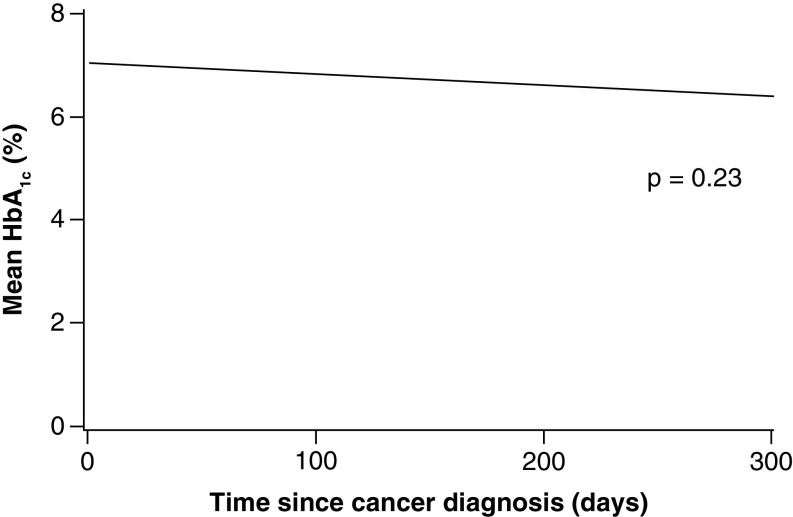
Mean hemoglobin A_1c_ levels within 1 year after leukemia diagnosis in patients with diabetes (n = 25). HbA_1c_ level did not change over time (p = 0.23). HbA_1c_: Hemoglobin A_1c_.

**Figure 2.**
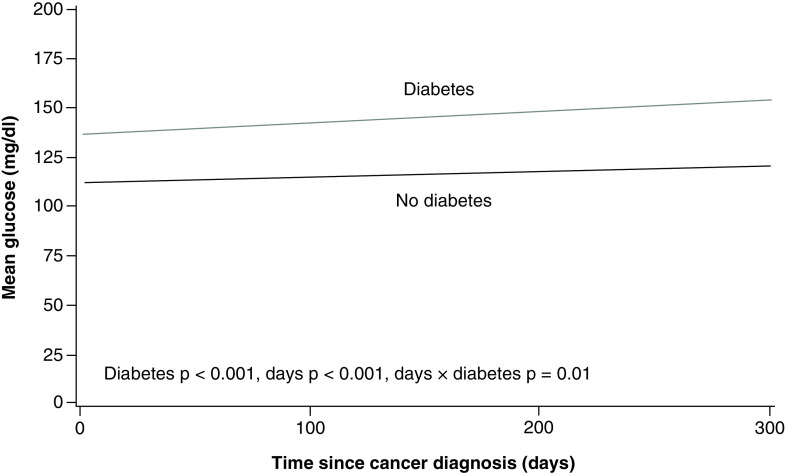
Mean glucose levels within 1 year after leukemia diagnosis for both groups. At the end of 1 year, mean glucose level was significantly higher in the diabetes group, but the mean glucose levels for both groups remained less than 180 mg/dl (fixed effects: diabetes, p < 0.001; days, p < 0.001; days × diabetes, p = 0.01).

Among patients with diabetes, 25 had at least 1 HbA_1c_ value measured within the year after leukemia diagnosis. For these patients, the mean (standard deviation) value was 6.8% (1.3%) and HbA_1c_ values did not change over time (Figure 1). Mean glucose values within the year after leukemia diagnosis were significantly different between patients with and without diabetes (p < 0.001) (Figure 2). There was a significant interaction effect (days × diabetes group; p = 0.01); glucose values increased in the diabetes patients during the year after diagnosis but remained stable in those without diabetes (Figure 2). Among 46 patients with data available on diabetes treatment, the largest percentage was treated with oral medication alone (n = 20, 43%), followed by insulin (n = 16, 35%) and diet modification (n = 7, 15%).

#### Diabetes effect on leukemia survival

**Figure 3.**
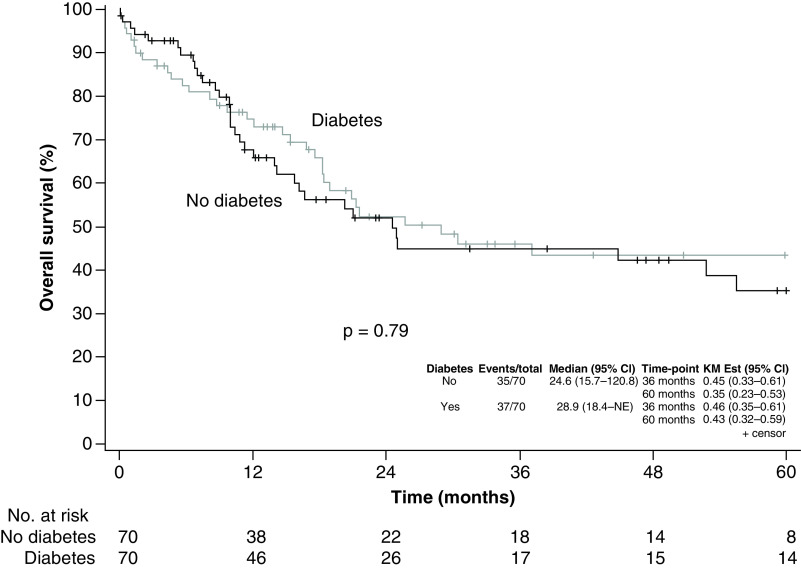
Kaplan–Meier curves estimating overall survival among patients with leukemia. There was no difference in survival between patients with and without diabetes (p = 0.79). KM: Kaplan–Meier estimate; NE: Not estimated.

**Figure 4.**
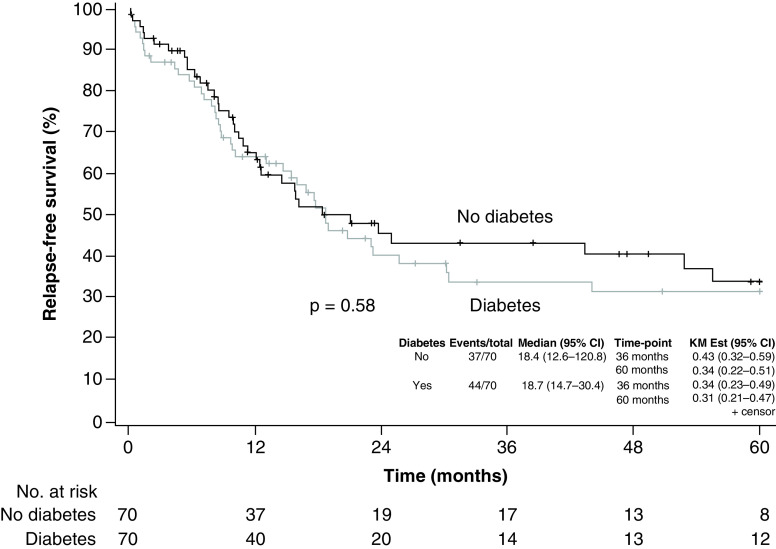
Kaplan–Meier curves estimating relapse-free survival among patients with leukemia. There was no difference in relapse-free survival between patients with and without diabetes (p = 0.58). KM: Kaplan–Meier estimate.

Median follow-up time was 23.2 months for surviving patients (Figure 3). The 3-year survival was estimated at 46% (95% CI: 35–61%) for diabetes patients versus 45% (95% CI: 33–61%) for patients without diabetes (p = 0.79). The hazard ratio (stratified for matched pairs) was 1.05 (95% CI: 0.57–1.94; p = 0.88). The 3-year relapse-free survival was estimated at 34% (95% CI: 23–49%) for diabetes patients versus 43% (95% CI: 32–59%) for patients without diabetes (p = 0.58) (Figure 4). The hazard ratio (stratified for matched pairs) was 1.10 (95% CI: 0.61–1.98; p = 0.76).

#### Diabetes effect on leukemia subtype in CML & AML

The majority of patients had AML (88.6%) (Table 1). Among the 16 patients with CML, the vast majority were in the chronic phase (bone marrow and peripheral blood contain <10% blasts, compared with blast phase in which bone and blood contain >20% blasts) (data not shown). Allelic variation status was also compared in patients with AML. The percentage of patients with variations and/or translocations was significantly higher in the AML group with versus without diabetes (81 vs 46%; p = 0.001), although more patient data were missing among diabetes patients. For AML cases risk-stratified on the basis of cytogenetic and molecular features into favorable, intermediate and poor subtypes, patients with diabetes had a lower prevalence of the intermediate subtype (17 vs 45% for no diabetes) and a higher prevalence of the unfavorable subtype (69 vs 40% for no diabetes; p = 0.01). Again, more patient data were missing among diabetes patients.

## Discussion

In this retrospective case–control study, diabetes was not associated with a decrease in OS in patients with AML or CML. This should be reassuring to hematologists and endocrinologists who treat patients with AML or CML and diabetes. One possible mechanism for this finding may be the potential protective effects of oral diabetic medications such as metformin, which have shown anticancer properties [18,19]. Interestingly, the majority of diabetes patients were taking oral medication alone for their diabetes. Our data did not distinguish specifically which oral medication was used, but given that metformin is first-line oral therapy for diabetes, it is likely that the vast majority of these patients were taking metformin when these data were collected. In addition, most patients in our study were not taking insulin at the time of leukemia diagnosis. Patients with cancer require close follow-up with their hematologists/oncologists, including regular laboratory measurements that are required to monitor their disease. Therefore, grossly abnormal serum glucose levels can be identified and managed early. Corticosteroids are commonly used in cancer treatment regimens, and early referral to an endocrinologist may be common if a patient’s hematologist/oncologist anticipates improved glycemic control before beginning chemotherapy regimens. Another explanation for these findings may be the use of insulin in diabetes patients during leukemia treatment. A recent study in a leukemic mouse model [20] showed that increased levels of insulin-like growth factor binding protein 1 (IGFBP1), an adipokine produced in adipocytes, were associated with insulin resistance and that giving these mice insulin decreased serum IGFBP1 levels.

Leukemia and its treatment also did not appear to affect glycemic control on the basis of HbA_1c_ levels, although the sample size was small. It is unclear why HbA_1c_ levels were not assessed in the majority of diabetes patients with leukemia during the year after leukemia diagnosis; this is an area that should be targeted for quality improvement. Although glucose values increased in patients with diabetes during the year after diagnosis, mean glucose values were not excessively increased among those with diabetes (<180 mg/dl). Glucose values in this range are most likely acceptable for patients undergoing active treatment for leukemia because intensive glycemic control in patients with diabetes undergoing treatment for leukemia is associated with worse outcomes [21]. Interestingly, patients with diabetes were more likely to be treated with corticosteroids than were controls. The reason for this is unclear, however and this observation should be interpreted cautiously.

We also found higher rates of allelic variations/translocations in diabetes patients with AML and a higher incidence of unfavorable cytogenetics among patients with diabetes. Studies evaluating allelic variation status in cancer and diabetes are lacking, particularly in patients with leukemia. One recent study evaluated somatic allelic variations in pancreatic cancer patients with and without diabetes and found a positive association between diabetes duration and somatic variation burden [22]. They also found novel (but infrequent) variations in the diabetes group. Future studies should specifically investigate allelic variational status in patients with leukemia with and without diabetes.

This study had some limitations. The sample size was small, the study duration was short and some relevant data were missing. This study also included a broad study population of patients with AML and CML and patients who had different forms of diabetes. A greedy matching algorithm was used but was limited to age and sex for matching. Other important variables could have been included for matching, but case numbers of patients with diabetes were small. Future studies with larger sample sizes should allow for more granular comparison between these subgroups – a larger prospective study would be ideal. Not all information regarding patient leukemia subtype status was available from the retrospective review. In addition, the majority of the cohort was non-Hispanic, which may limit the applicability to other racial and ethnic groups. A large percentage of patients with diabetes did not have HbA_1c_ values available, because the focus was most likely on cancer treatment. The increase in glucose values also may have resulted from the focus on cancer treatment. Increasing frequency of monitoring of HbA_1c_ values and glucose control represents a potential area of quality improvement.

## Conclusion

In this case–control study, diabetes mellitus was not associated with a decrease in OS in patients with AML/CML. Moreover, AML/CML did not affect glycemic control.

## Future perspective

Despite the study limitations, and similar to findings from most analyses of solid organ cancers, coexisting diabetes was not associated with an increased mortality rate in patients with leukemia. There are limited studies addressing long-term outcomes in patients with leukemia and diabetes; confirmation of these observations in larger patient cohorts is needed. Investigation of the effect of intensive insulin therapy on leukemia survival rate is of interest and worthy of future study.

**Summary points**

- In this retrospective case–control study, diabetes was not associated with decreased survival in patients with leukemia (acute myeloid leukemia and chronic myeloid leukemia).
- Hemoglobin A_1c_ values did not change over time in diabetic patients with leukemia.
- Mean glucose values were significantly different between patients with and without diabetes, but mean glucose levels did not exceed 180 mg/dl.
- Leukemia was not associated with poor glycemic control.
- Among patients with acute myeloid leukemia, the number of allelic variations and/or translocations was significantly higher in those with diabetes.
